# Facile Recoverable and Reusable Macroscopic Alumina Supported Ni-based Catalyst for Efficient Hydrogen Production

**DOI:** 10.1038/s41598-019-52857-4

**Published:** 2019-11-08

**Authors:** Siow Hwa Teo, Davin Kin Yew Yap, Nasar Mansir, Aminul Islam, Yun Hin Taufiq-Yap

**Affiliations:** 1 Catalysis Science and Technology Research Centre, Faculty of Science, Universiti Putra Malaysia, 43400 UPM Serdang, Selangor Malaysia; 2 Department of Chemistry, Faculty of Science, Universiti Putra Malaysia, 43400 UPM Serdang, Selangor Malaysia; 3 Chancellery Office, Universiti Malaysia Sabah, 88400 Kota Kinabalu, Sabah Malaysia; 4 Department of Petroleum and Mining Engineering, Jashore University of Science and Technology, Jashore, 7408 Bangladesh

**Keywords:** Chemistry, Catalysis

## Abstract

A *γ*-NA5 catalyst in the form of pellet was first to be reported and was pioneering in gasification to accelerate the production of syngas through biomass (palm empty fruit brunch) conversion. The synthesised *γ*-NA5 pellet possesses a high surface area of 212.32 m^2^ g^−1^, which renders more active sites for hydrocarbon cracking, subsequently leading to high H_2_ production (0.0716 m^3^ kg^−1^). Additionally, the pellet exhibits remarkable reversibility and reusability with 91% H_2_ production efficiency being retained after five consecutive gasification cycles. Distinctively, the feature of the synthesised *γ*-NA5 pellet from the conventional powder-like catalyst is that it eases the separation of the used catalyst from the biomass ash, and subsequently facilitates regeneration solely by calcination process. The loading of 20 wt.% optimised amount of catalyst itself has successfully shorten the completion of gasification process up to 135 min, which is highly feasible for a large scale industrial usage after considering the cost of the catalyst, facile preparation method, and catalyst’s effectiveness towards gasification.

## Introduction

Relying solely on solar power is insufficient to maintain a clean and sustainable energy environment. Along with the progression towards green energy move, the generation of clean energy from biomass conversion or biomass gasification is literally important to plunge the use of non-renewable fossil fuels which is the cause for air pollution and greenhouse effect^1^. To date, biomass is still an important energy resource for cooking and heating in developing countries in Asia and Africa. The conversion of biomass into hydrogen or hydrogen-rich gas indeed offers a research competitive means to produce energy and chemicals from low-cost renewable sources^2^, which is highly applicable around the globe, typically for under-developed and developing countries. Generally, gasification techniques are the most suitable routes to recover energy from biomass waste^3^. Biomass gasification is performed at a high temperature by partially oxidising the carbonaceous contained in the biomass feedstock for hydrogen production with the usage of a controlled amount of gasifying agents, such as air, pure oxygen, or steam^4,5^. During the gasification process, the biomass structures breakdown under a suitable thermal treatment by generating a mixture of gaseous substance with minute amounts of char, oil, and ash by-products according to the reaction mechanism below^6^ :

$${\rm{Biomass}}+{\rm{heat}}+{\rm{oxidant}}\,({{\rm{O}}}_{2},{{\rm{H}}}_{2}{\rm{O}})\to {{\rm{H}}}_{2}+{\rm{CO}}+{{\rm{CO}}}_{2}+{{\rm{CH}}}_{4}+{\rm{Tar}}+{\rm{Char}}+{\rm{Ash}}$$

To ensure an effective catalytic gasification process, the employment of catalysts during the gasification process (so-called catalytic gasification) is expected to reduce undesirable tar formation^7^ and reduce reaction temperature^8^. Most studies agreed that the activity of an alkali metal is proportional to its atomic weight, whereby the alkali metal’s activity increases with the increasing atomic weight from lithium to cesium^9^. Matsukata *et al*.^10^ reported that an optimum loading of 3% to 3.5% of potassium is essential for the formation of petroleum coke, while >5 wt% is for the formation of bituminous coal^11^. Nevertheless, one major problem of potassium catalysed gasification is the volatility of the potassium species, whereby the released gas-phase KCl leads to gasifier corrosion^12^ and toxic vapours^13^. Meanwhile, the sodium may cause the formation of catalytically-inactive sodium aluminosilicates, attributed to the undesirable evaporation and reaction between sodium and other mineral matter^14,15^. The abundancy, cost-effectiveness, and non-volatile properties of the calcium compounds (CaCO_3_, CaO, and Ca(NO_3_)_2_) > 950 °C has attracted a number of researchers. However, they are deactivated during the high temperature sintering process, and thus impeding its application in the industrial scale^16,17^.

A number of heavy metallic catalysts i.e., Pd (Palladium)^18^, Ru (Ruthenium)^19^, Pt (Platinum)^20^, Cu (copper)^21^, Fe (iron)^22^, and Ni (nickel)^23,24^, have been reported to enhance the catalysed gasification reactions, tar reduction, conversion efficiency improvement, and to promote the purity of syngas^25,26^. Evidently, Claude *et al*.^27^ demonstrated that nickel is a promising gasification catalyst for downstream tar reforming owing to its efficient production of H_2_ and CH_4_^28^ and cheap by-product of hydrotreating^29^. However, the single bulk Ni catalysts were suffering from rapid deactivation due to the formation of undesirable coke^30^, which calls for the modification of Ni catalysts with other metal supports^31,32^. In 1997, Simell *et al*. reported Al_2_O_3_ as the most suitable support for Ni catalysts, credited to its chemical and physical stability and high mechanical properties^33^. Subsequently, Wu and William revealed that the Ni-Al (1:2) catalysts which was prepared through co-precipitation demonstrated excellent catalytic abilities of high H_2_ production (64vol.%) and the synergism between Al and Ni plunged the coke formation^34^, which was consistent with other reports of Ni-Al catalysts biomass gasification^35–38^.

Conclusively, the addition of Al within the Ni-catalyst framework has effectively improved the catalytic performance of the gasification reaction. However, the prepared Al-Ni catalysts to date are in fine powders (i.e. mm in diameter that offer high catalytic activity), which render several drawbacks, such as high pressure drops, poor mass/heat transfer, and poor contact efficiency when it is treated in a large volume of gases^39,40^, and possible health risks caused by inhalation of small particles. Interestingly, we discovered that the pressure drop could be mitigated by almost two orders of magnitude when we shaped the catalysts in macroscopic spherical form. It is expected that the application of macroscopic sphere catalysts in gasification reactions could ease the separation of catalyst from residue of biomass and the catalyst is easy to handle and recyclable for several times, which are the aims of this research.

In this research, the catalytic performances of a *γ*-NA5 catalyst (the doping of Al in Ni catalyst lattice) was investigated, in the form of pellet for biomass (palm empty fruit brunch) gasification. The novelty of this study is that our synthesis macroscopic spheric *γ*-NA5 catalyst possesses more active sites for hydrocarbon cracking with high H_2_ production. The pellet exhibited excellent reversibility and reusability for gasification process. More importantly, our synthesised *γ*-NA5 pellet could separate the used catalysts from the biomass ash with ease and facilitate the regeneration process solely by calcination process. In addition, the speed-up gasification process of the *γ*-NA5 catalyst in a self-customised gasifier is highly feasible for large scale industrial usage after considering the cost of the catalyst, facile preparation method, and catalyst’s effectiveness towards gasification.

## Results and Discussion

**Scheme 1:**
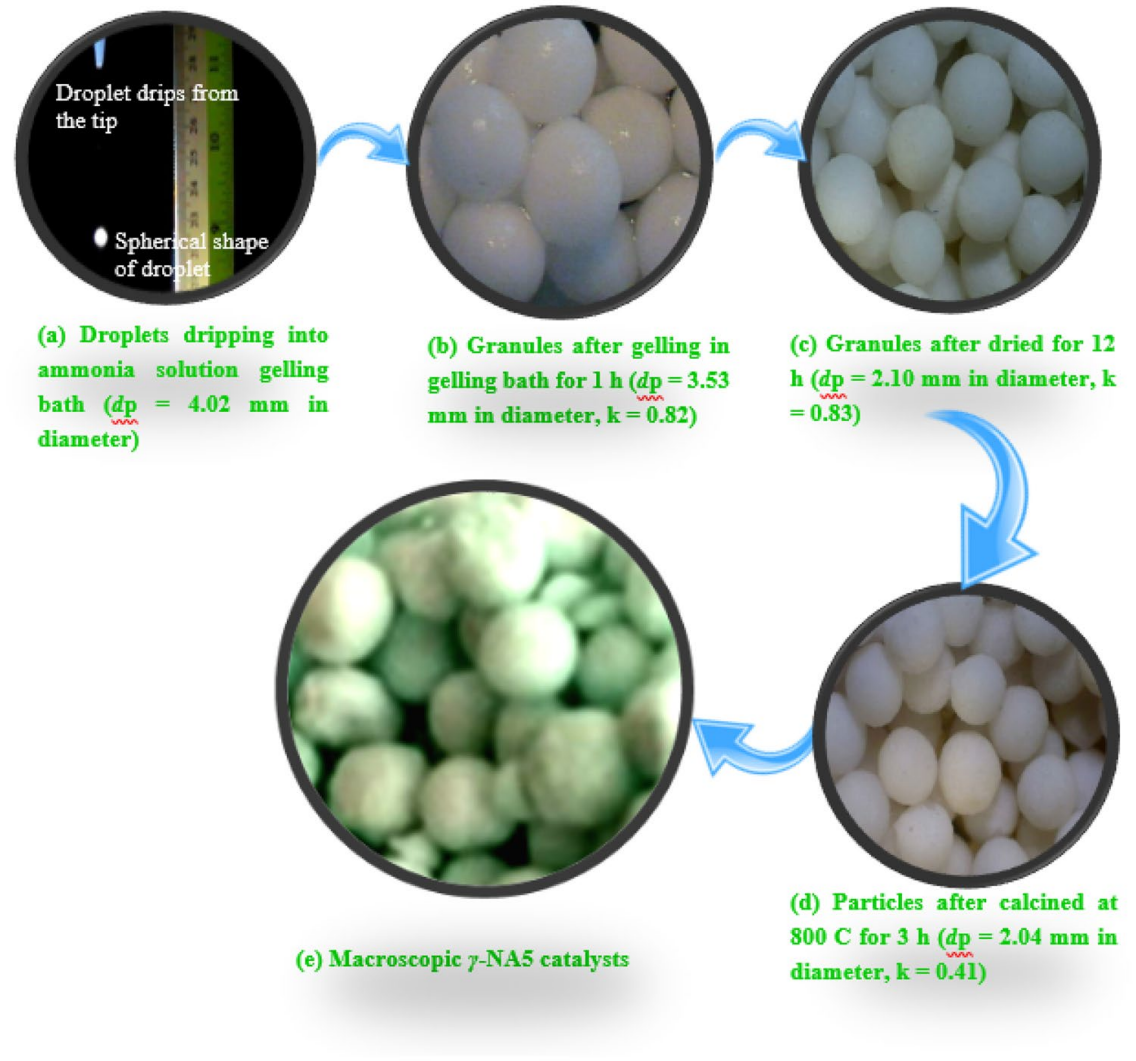
Stepwise particles preparation (for macroscopic spherical *γ*-NA5 catalysts).

The macroscopic spherical *γ*-NA5 catalysts derived from sol-gel process after calcination is schematically presented in Scheme 1. For gelling capability analysis, the gelling property of the aluminium oxide hydroxide (AlOOH) solution was studied and revealed in Fig. S1. A clear settling region could be seen at pH values ≥ 4.0, while a better homogenous gelling phase at pH ≤ 4.0. Philipse *et al*.^41^ who studied the durability of suspension of boehmite silica mixture recommended that a stable suspension could be formed under acidic condition owing to the attraction force between particles. Hence, the absence of settling at pH 2 and 1 (Fig. S1) could be attributed to an enhance in density due to flocculation effect between particles. In addition, the increase in viscosity could be one of the reasons for settling issue, as accordance to Stoke’s law. In brief, the lower the pH of the suspension enables the primary particles in the suspension to attract one another and are then likely to form gel. Finally, this gel formed at pH 2 (ideal pH value) was used for the formation of macroscopic particles.

The preparation of macroscopic Al_2_O_3_ supported beads was initiated by dripping granules from the tip of a needle, in conjunction with the formation of spherical particles during the dripping process (Fig. S2). The size of AlOOH droplets that dropping from the tip of the needle, possessed 4.02 mm in an average diameter (Scheme 1a). Upon gelation for 1 h, concentrated AlOOH granules (Scheme 1b) were become smaller as compared to the size of the fresh droplet dripping from the tip of the needle, which formed the shinning particles with 3.53 mm in average diameter and shrinkage of 10%. This process was corresponded with removal of solvent and water from the gel matrix (syneresis)^42^, hence, reduced the volume of the particle. The AlOOH granules contracted extensively upon air drying for 12 h (Scheme 1c), achieved significant average diameter and shrinkage of 2.10 mm and 46%, respectively. As manifested in Scheme 1d, the particles were converted into hard agglomerated particles due to formation of solid bridge. Finally, the AlOOH granules were shrunk more extensively to maximum size of 2.04 mm (size reduced by 50%) upon calcination at 800 °C (Scheme 1d). From the size measurement model, it is to noted that the calcined particles with 0.04 ± 0.01 of sphericity factor, showing that the *γ*-Al_2_O_3_ particles were spherical in shape.

**Scheme 2:**
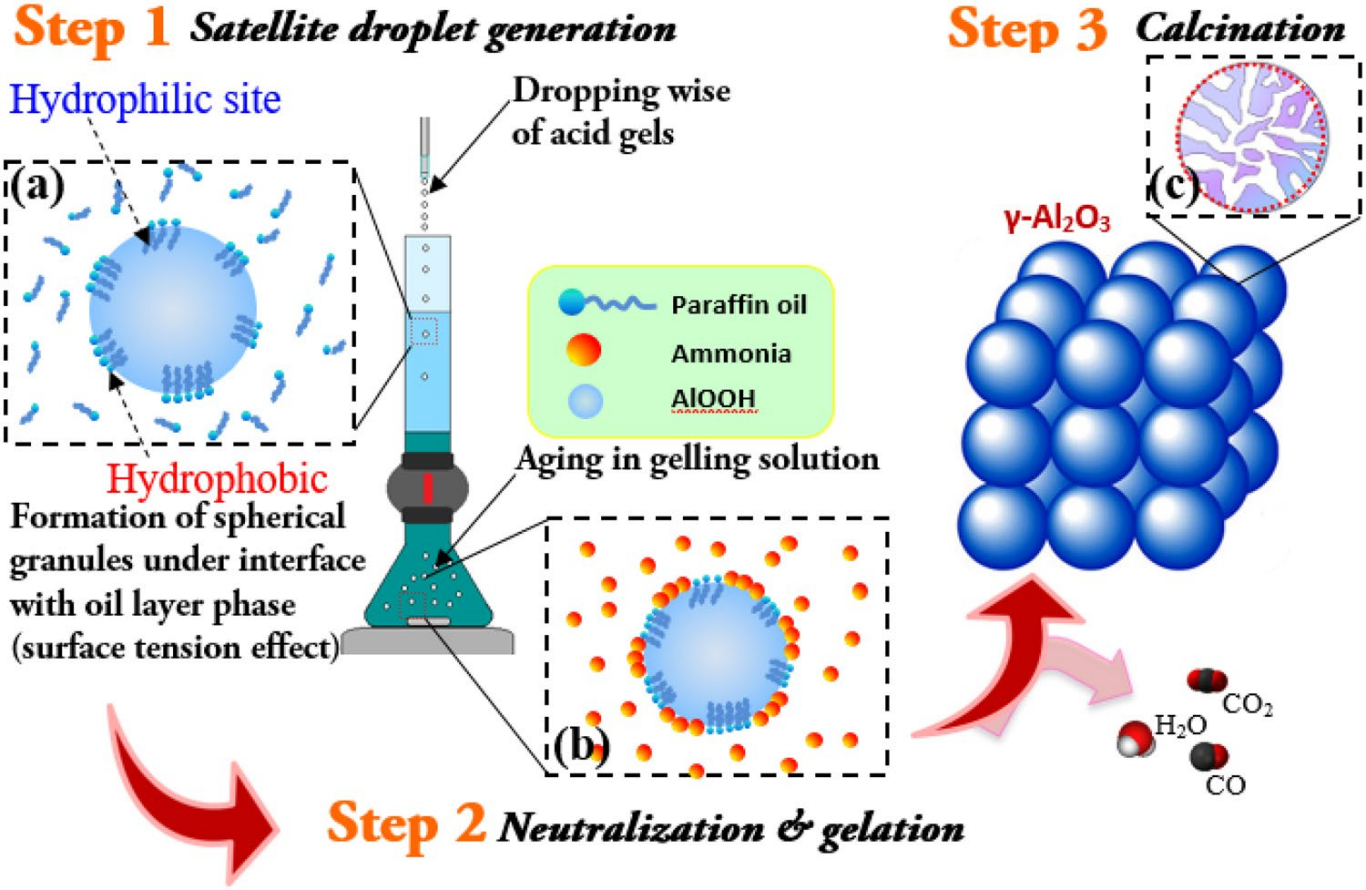
The proposed growth mechanism of spherical alumina granule particles.

The proposed growing mechanism of alumina was presented in Scheme 2. Step 1 is the formation of boehmite droplets on the oil surface due to high surface tension, followed by the aging of gel droplets in the ammonia solution (Step 2) for 1 h. Initially, three main scenarios are involved in the development of AlOOH droplet formation, which are the extension of the gel protrusion, the disconnection of the gel ligament, and the accomplishment of the gravity droplet generation. Scheme 1a designates the shape of the droplet remained almost spherical shape through the droplet formation route. It is notable that the formation of these spherical particles is closely dependent on the distance between the tip of the needle and level of silicon oil. In 1966, Taylor *et al*.^43^ reported that, at the liquid/air interface, the liquid droplets (water/oil) would retain their shape in spherical under a specific combination of liquid properties (viscosity and surface tension). Subsequently, it will continues shifted to downward and away from surface of oil layer. When AlOOH droplet solution came in interaction with paraffin oil layer phase, hydrophobic and hydrophilic interactions occur instantaneously between AlOOH – paraffin (water – oil) interfaces (Scheme 2a). Paraffin contains both a hydrophilic head group and a hydrophobic tail. The hydrophilic head moiety avoids phase separation of the AlOOH droplet molecules by upholding the hydrophilic moiety via formation of strong hydrogen bonds with AlOOH droplet molecules. Some water was squeezed out from the droplet molecules and resulting in tightening of particles. After finishing the process, aging of the wet AlOOH granules further happened when the granules were contacted with alkali solution (pH > 7).

In step 2 (Scheme 2b), ammonia would neutralise the acid within the wet gel granules, which resulted in the formation of rigid wet-gel droplets. Subsequently, the particles were isolated by simple physical filtration, washed thoroughly with deionised water, and dehydrated at ambient condition. Finally, alumina phases were then calcined at >700 °C (Step 3) and expected to undergo a phase transformation to *γ*-Al_2_O_3_. In addition, the interconnection of mineral *γ*-Al_2_O_3_ framework was constructed as further water was eliminated through a condensation process and hence, forming a mesoporous/macroporous material with cylindrical pore structures (Scheme 2c).

**Scheme 3:**
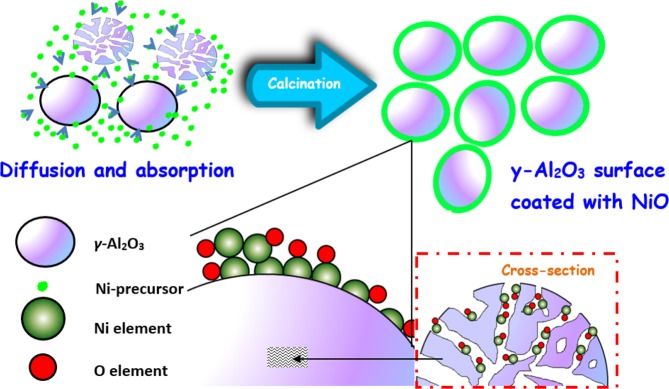
The proposed growing mechanism of macroscopic spherical *γ*-NA5 catalysts

Upon doping the synthesised alumina with a Ni precursor by incipient wetness impregnation method, the greenish macroscopic sphere, denoted as *γ*-NA5 catalyst, was then calcined at 500 °C, as presented in Scheme 3. In this study, porous *γ*-Al_2_O_3_ particles formed after calcination at 800 °C was used for catalyst support. As a result of calcination effect, pores were formed from the void spaces inside the alumina particles upon the elimination of water from the crystal planes of the *γ*-Al_2_O_3_^44^. Consequently, nickel ions were diffused into meso- and macroporous porous structure of *γ*-Al_2_O_3_ support and attached to the inert wall of pores during impregnation process. In addition, the impregnation procedure consisted of preparing samples with Ni loadings by incipient wetness impregnation of the *γ*-Al_2_O_3_ supports with aqueous solutions of NiCl_2_•6H_2_O of appropriate concentrations (typically 5 wt%). The solution pH’s was kept at 8. In a colloid preparation, the colloid stability is a principal parameter that influences by the change of solution pH. A very high at pH above 9, the reduction rate of particle is slow resulting an aggregation of nanoparticles^45^. A conclusion has been reported by Eskandari *et al*.^45^ that the formation of nanoparticle can be defends owing to the strong electrostatic repulsion under the pH 8. Hence, pH of the solution was kept at 8 in this study in order to synthesise NiO nanoparticles onto the *γ*-alumina supports to produce macroscopic spherical *γ*-NA5 catalyst. The prepared macroscopic spherical *γ*-NA5 was coated with NiO and the active components of catalyst were very tiny irregular particles coated on the surface of support with a size range of 12–26 nm (Fig. 2e). The newly developed catalyst exhibited a high BET surface area of 212 m^2^ g^−1^ (Table 1), which was about 29-fold larger in surface area than the commercial nickel-based catalyst. This implied its feasibility as an efficient catalytic material.

**Figure 1:**
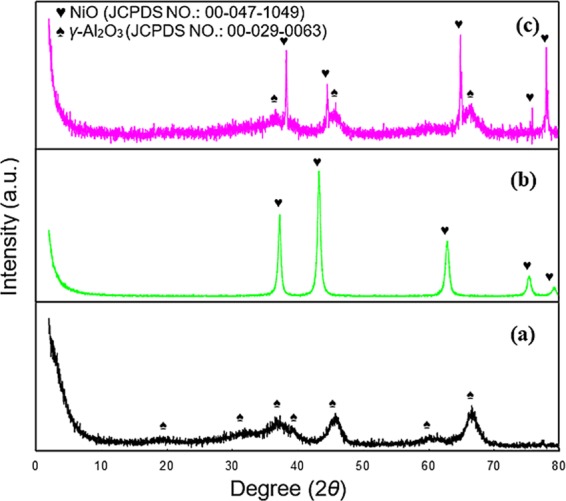
XRD patterns of *γ*-Al_2_O_3_ (**a**), NiO (**b**) and macroscopic spherical *γ*-NA5 (**c**) catalysts.

**Figure 2:**
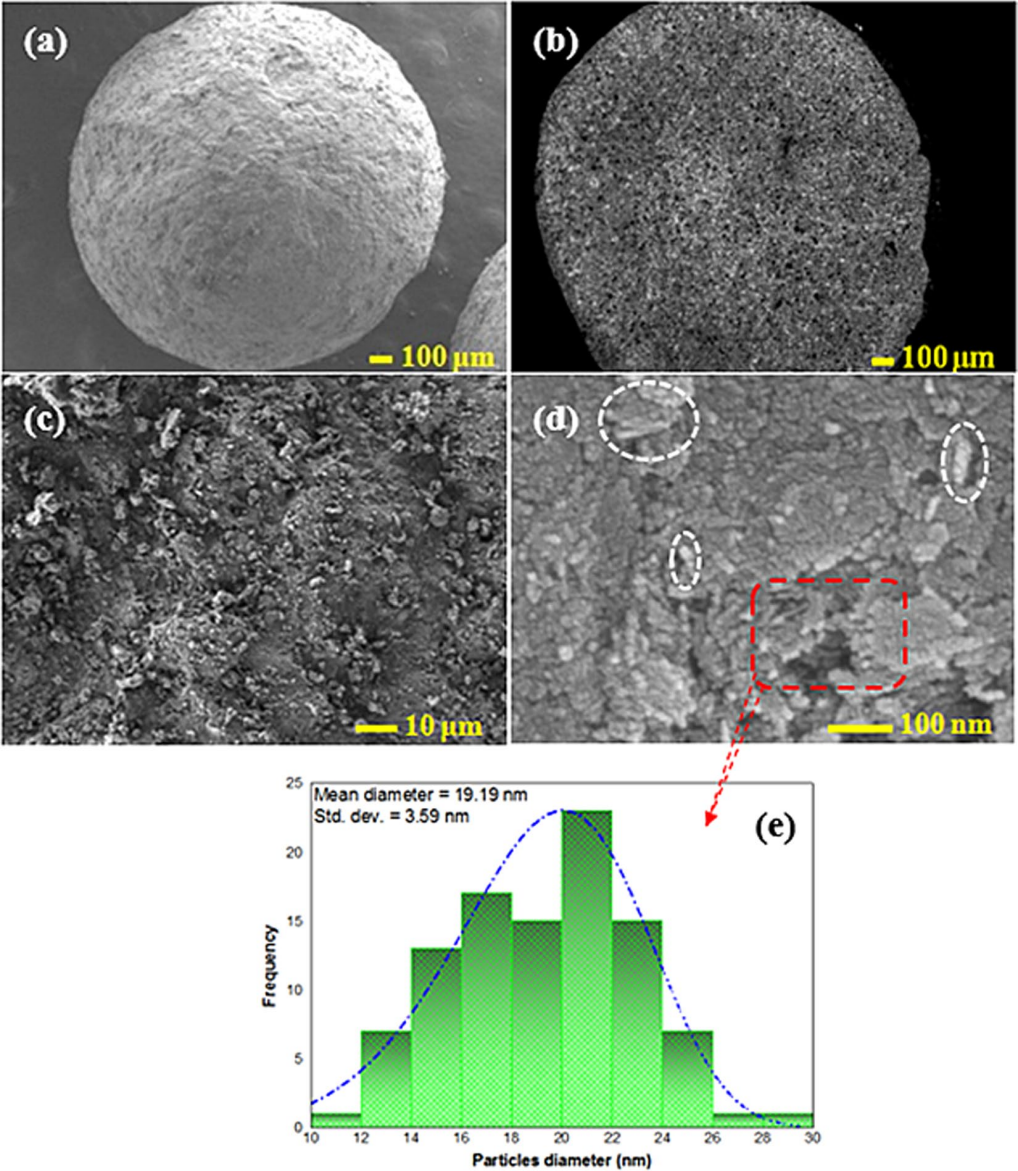
SEM images of *γ*-Al_2_O_3_ (**a**), cross-sectional view of *γ*-Al_2_O_3_ (**b**) surface view of *γ*-Al_2_O_3_ (**c**), and macroscopic spherical *γ*-NA5 (**d**) catalysts; and particles distribution of NiO (**e**).

**Table 1:** Particle size, BET surface area, pore volume, pore diameter, TPR-H_2_ and H_2_ yield analysis of catalysts.

Catalysts	Granule size (mm)	Surface area^a^ (m^2^ g^−1^)	Pore volume^a^ (cm^3^ g^−1^)	Pore diameter^a^ (nm)	Reducibility^b^ (μmol g^−1^)	Yield of H_2_^c^ (m^3^ kg^−1^)
**Metal oxide**						
*γ*-Al_2_O_3_	2	237.17	1.40	22.2	218.16	0.0128
NiO	—	7.29	—	—	1215.62	0.0697
**Supported catalyst**						
*γ*-NA5	2	212.32	0.63	6.4	2118.94	0.0716

^a^BET surface area.

^b^H_2_-temperature programmed reduction.

^c^Calculated from MS analysis.

The crystallinity of the synthesised catalysts (NiO, *γ*-Al_2_O_3_, and *γ*-NA5) were determined through XRD, as depicted in Fig. 1. Firstly, at a high calcination temperature of 700 °C, it was expected that the alumina phases had completely transformed to *γ*-Al_2_O_3_ catalysts (Fig. 1a); with highly crystalline peaks observed at 19.5°, 31.9°, 37.6°, 39.5°, 45.8°, 60.5°, and 66.7°. The experimental peaks closely matched the theoretical values from JCPDS File No. 00–029–0063 and were consistent with other reported works in literature^46^. Secondly, we investigated the crystallinity of NiO, as shown in Fig. 1b, whereby the bulk NiO in the form of powder (JCPDS file No. 00-047-1049) exhibited highly crystalline peaks at 37.3°, 43.3°, 62.9°, 75.3°, and 79.4°, indicating the formation of a purely cubic NiO.

Successively, the prepared macroscopic *γ*-NA5 catalyst (Fig. 1c), possesses both the characteristic peaks of *γ*-Al_2_O_3_ (2*θ* = 37.6°, 45.8°, and 66.7°) and NiO (2*θ* = of 37.3°, 43.3°, 62.9°, 75.3° and 79.4°), indicating the well-embedment of NiO within the *γ*-Al_2_O_3_ catalyst. Apart of that, a noticeable shift of NiO peaks of macroscopic *γ*-NA5 catalyst (Fig. 1c) was discerned compared to that of pure NiO (Fig. 1b). The shift in peaks and change in intensity of the peaks may be due to the change in bond length due to bond stretching of the mixed metal oxide catalyst. Paumier *et al*.^47^ speculated that Bragg angles may shifts in both directions for the same sample for different Bragg peaks, which might be attributed to the residual stresses and strain originated in the particles. It has been shown by Seifried *et al*.^48^ that structural and/or microstructural changes in the particle may change the Bragg peaks, but no conclusive evidence for this conjecture has yet been provided in the literature. However, all the peaks associated with the macroscopic *γ*-NA5 catalyst was indexed with JCPDS file no. of 00-029-0063 and 00-047-1049. The complete formation of macroscopic *γ*-NA5 catalyst (Fig. 1c) was evident through the weakening intensities of the *γ*-NA5 peaks upon the introduction of NiO, which proved the formation of macroscopic *γ*-NA5 catalyst. Additionally, the XRD profile showed absence of additional crystalline peaks (with no formation of new mixed oxide phases), suggests that high purity binary system solid oxides were successfully synthesised.

The topographic morphology and cross-sectional area of the *γ*-NA5 catalyst are manifested in Fig. 2. A rigid and rough pristine *γ*-Al_2_O_3_ catalyst surface was presented, as shown in Fig. 2a,c. Interestingly, the alumina sphere was highly porous, in which the interior of the alumina sphere was engineered with highly interconnected hierarchical pores (Fig. 2b). It was hypothesised that the high porosity of *γ*-Al_2_O_3_ indicated the accessibility and permeability of reactant gaseous within the active phases of *γ*-NA5 catalyst, even after the impregnation of NiO.

Meanwhile, morphological alteration was observed where a tiny and irregular grain-like macroscopic *γ*-NA5 structure was formed when NiO particles were deposited on the alumina surface (Fig. 2d). It was speculated that spherical NiO particles (average particle size of 19 nm, Fig. 2e) could fill the porous channels during the formation of *γ*-NA5, credited to the ability of *γ*-Al_2_O_3_ in reducing the particle size of NiO to some extent. It was assured that the impregnation of NiO, followed by the calcination process at 500 °C, produced the NiO in an average particle size of 19 nm. This small particle size of NiO could still accord for a porous network, which enhanced catalytic reaction to take place. Nevertheless, we also observed the inhomogeneity on the *γ*-Al_2_O_3_ surface, whereby some of the small granules (white circle) were crystallised on the *γ*-Al_2_O_3_ surface when the active sites of *γ*-Al_2_O_3_ were fully occupied (Fig. 2d). In brief, the synthesised highly porous pelleted catalyst offers a large and accessible surface area for biomass gasification reaction and was expected to suppress back pressure drop in flow gasifier.

**Figure 3:**
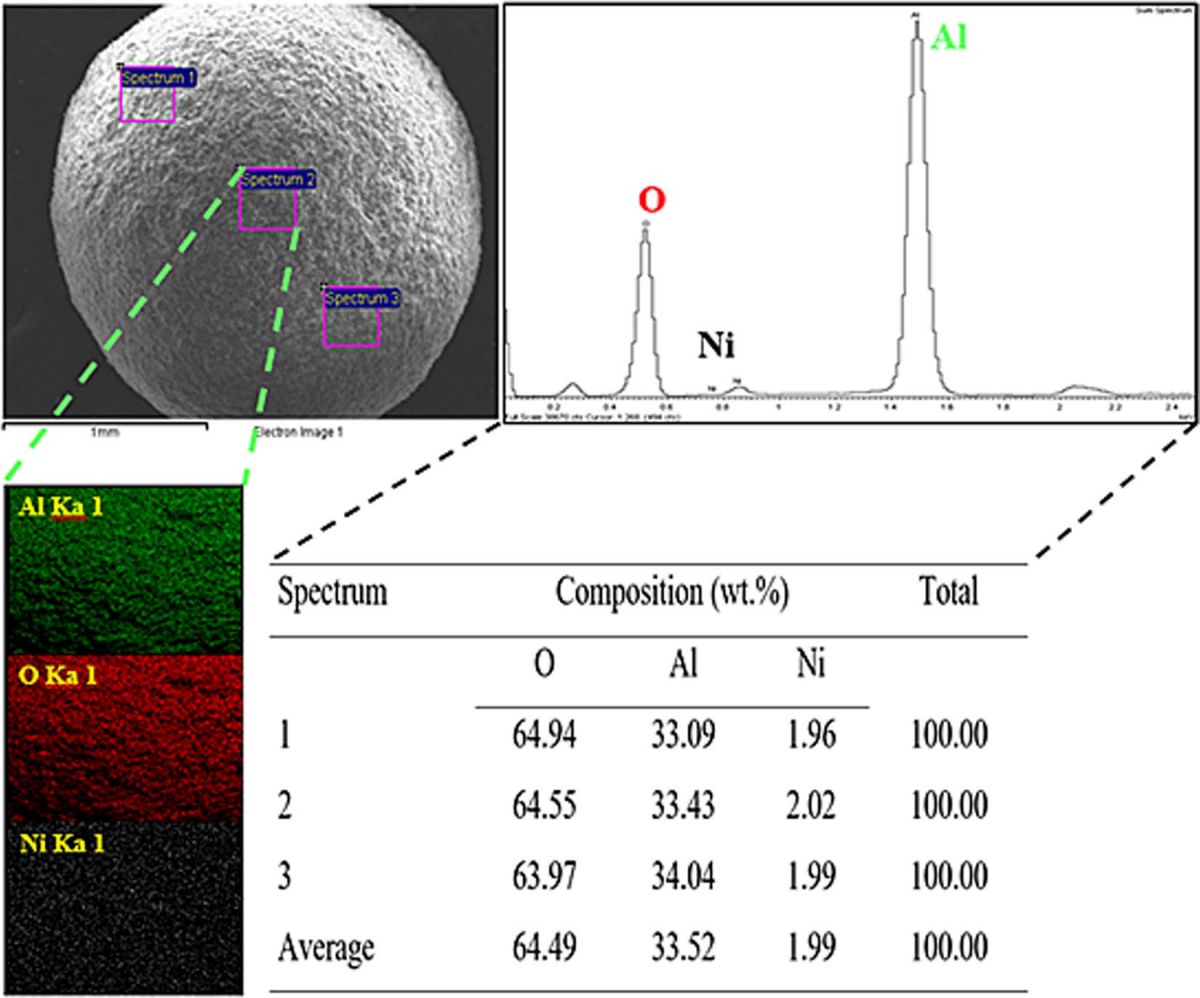
SEM-EDX mapping and elemental composition analysis of macroscopic *γ*-NA5 catalyst.

The elemental composition and mapping analysis of the synthesised macroscopic *γ*-NA5 catalyst was tested using SEM-EDX measurement (Fig. 3). The macroscopic *γ*-NA5 comprised of elements aluminium (Al), oxygen (O) and nickel (Ni), as presented by the diffractogram and EDX mapping results. According to the results of EDX elemental analysis (Table), it demonstrates that the chemical compositions of macroscopic *γ*-NA5 catalyst, which was expressed as weight percentage (wt.%). The composition of the Al was measured to be approximately 33.04 - 33.43 wt.%. Meanwhile, the average content of the Ni calculated from three tested spectrums showed 1.99 wt.%. Ni element in macroscopic *γ*-NA5 catalyst was significant owing to the homogenised on the surface of catalyst representing that the catalyst has a high dispersion^49^. Apart from that, Ni molecules was also diffused into meso- and macroporous catalyst structure of *γ*-NA5 and attached to the inert wall of pores during synthesis process (Scheme 3). Hence, the measured area was mostly packed with the Al and O elements, respectively.

**Figure 4:**
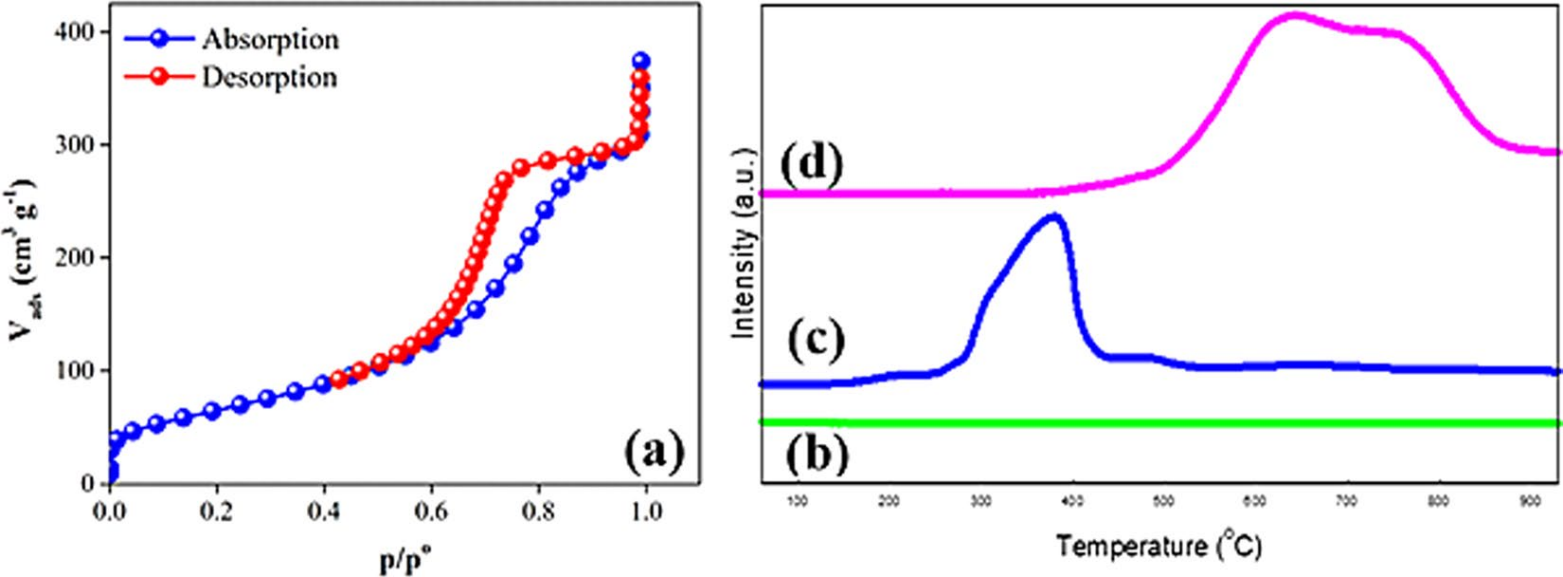
The N_2_-adsorption-desorption isotherm of the macroscopic *γ*-NA5 catalyst (a), (b) Temperature programmed reduction-H_2_ profiles of *γ*-Al_2_O_3_ (b), NiO (c) and *γ*-NA5 (d) catalysts.

The Type-IV nitrogen adsorption–desorption isotherms of the *γ*-NA5 catalyst (in accordance to the IUPAC classification) were explicated in Fig. 4a, indicates the formation of mesoporous *γ*-NA5 catalyst. The isotherms of *γ*-NA5 catalyst revealed H2-typed hysteresis loops (both the adsorption and desorption processes), whereby the two branches remained closely vertical and parallel over an appreciable range of gas uptake at a relative pressure (*P/P*_0_) range of 0.4 to 1.0, indicating macropores (>50 nm) and mesopores (2–50 nm) fillings. According to the IUPAC classification the isotherm obtained in this study, the characteristic of adsorbents that are compatible to the type IV isotherm (Fig. 4a). The generated pore is narrow and depth which is a remarkable characteristic of mesoporous structure with a cylindrical pore structure. A similar observation was reported by Cychosz *et al*.^50^, who studied physio-adsorption properties of porous materials.

Table 1 summarises the total BET surface area, average pore diameters, and pore volumes of the granule particles *γ*-Al_2_O_3_, bulk NiO, and *γ*-NA5 catalysts. The calcined granule particles (*γ*-Al_2_O_3_) rendered a high surface area of 237 m^2^ g^−1^ after calcination at 800 °C (Table 1), attributed to the formation of void within the alumina particles upon the removal of water from the crystal planes of the aluminium oxide^44^, which was in great agreement with the SEM image. When NiO was loaded on the granule *γ*-Al_2_O_3_ particles, the surface area (212 m^2^ g^−1^) and pore volume (0.63 cm^3 ^g^−1^) were substantially reduced, suggesting the deposition of NiO catalysts within the *γ*-Al_2_O_3_ support which resulted in a partial reduction of the porous network. Barrett, Joyner, and Halenda (BJH) demonstrated the pore size distribution of the *γ*-NA5 from the adsorption branch of the isotherm (Fig. S3), which exhibited one single narrow-peak corresponding to the presence of unimodal distribution pores (monodisperse) of 2.2–5.1 nm in the particles. This indicated the formation of meso-pores, which encouraged the gasification process.

Temperature programmed reduction (TPR-H_2_) for *γ*-Al_2_O_3_, bulk NiO, and *γ*-NA5 catalysts is shown in Fig. 4b–d and the results are summarised in Table 1. TPR analysis of the pure *γ*-Al_2_O_3_ support catalyst (Fig. 4b) was included for comparison purposes. No H_2_ consumption peak was observed in TPR spectra for pure *γ*-Al_2_O_3_, indicating that there was no occurrence of reduction process on the *γ*-Al_2_O_3_ surface, due to the highly stable *γ*-Al_2_O_3_. The highly stable bulk *γ*-Al_2_O_3_ was credited o a strong O = Al-O-Al = O bond. In contrast, the pure NiO exhibited a reduction peak at 300 °C–400 °C (Fig. 4c), representing the reduction of Ni^2+^ to Ni^0^ at lower temperature. The synthesised *γ*-NA5 catalyst (Fig. 4d) exhibited a broad and highly intense reduction peak over the temperature range of 550 °C–850 °C. This redshifted *γ*-NA5 reduction peak of above 550 °C (Fig. 4d) could be due to the strong chemical interaction between nano-sized NiO dopant and the *γ*-Al_2_O_3_ catalyst support, which led to the occurrence of reduction process at high reduction temperature.

### Catalytic activity

**Figure 5:**
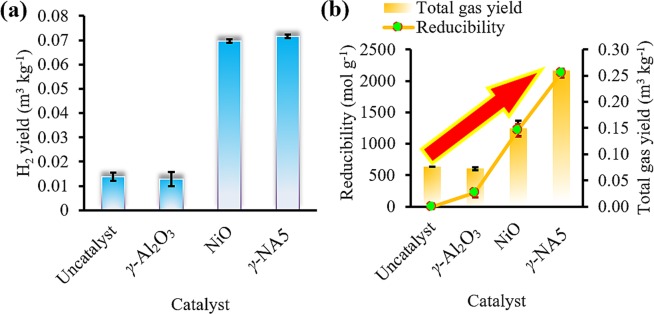
Effect of uncatalytic and catalytic (*γ*-Al_2_O_3_, NiO, and *γ*-NA5 catalysts) fast gasification process (**a**) and correlation of reducibility of catalysts with total gas yield production (**b**). [Reaction conditions: reaction temperature 900 °C, catalyst to biomass ratio 20 wt. % and flow rate 10 sccm].

H_2_ yield production of the gasification process either with catalyst (*γ*-NA5 catalyst, bulk *γ*-Al_2_O_3_, and bulk NiO) or without catalyst were demonstrated in Fig. 5a and the results were summarised in Table 2. From the results obtained, the bulk *γ*-Al_2_O_3_ and uncatalysed (Fig. 5a) flash gasification of palm empty fruit branch processes produced small amounts of H_2_ yields, which were 0.013 and 0.014 m^3^ kg^−1^, respectively. The minute amount of hydrogen production during flash gasification was attributed to low ash content within the biomass because biomass ash naturally consists of inorganic metal oxides, i.e. K and Na, which serves as catalysts for hydrogen production^51^. To be specific, the bulk *γ*-Al_2_O_3_ catalyst reaction rendered lower hydrogen production than the uncatalysed reaction, due to *γ*-Al_2_O_3_ inhibiting hydrogen production. Meanwhile, the bulk NiO exhibited higher H_2_ production yield of 0.0697 m^3^ kg^−1^. When NiO was added to *γ*-Al_2_O_3_ catalyst_,_ *γ*-NA5 catalysts (Fig. 5a) exhibited 1.03-fold increment in H_2_ yield of up to 0.0716 m^3^ kg^−1^, indicating its excellence in flash gasification catalytic performance, including tar cracking reaction. A number of Ni based powdered catalyst for biomass derived hydrogen production was reported in the literature including Ni/Ce/Al_2_O_3_^52^, Fe-Ni-Ru/γ-Al_2_O_3_^53^, NiO^54^, Ni/Pt/dolomite^54^, Ni/CeO_2_^55^, Ni/Al_2_O_3_^55^, Ni/MgO^55^, and Ni/MnO_2_^55^. The yield of H_2_ is comparable with the results reported in the literature though the experimental conditions of different catalysts are not identical. However, no powdered catalyst was reported the reusability of catalyst for hydrogen production. More importantly, the reusability of macroscopic catalyst obtained from this study was maintained high yield even after five times of successive reuse as demonstrated in Fig. 7. Comparatively, the lower H_2_ production by the pure NiO single metal system (Fig. 5a) was ascribed to a low specific surface area (proven in BET in Table 1) and severe coking process, which inhibited H_2_ production. Fig. 5b shows the order of reducibility in the sequence of *γ*-NA5 > NiO > *γ*-Al_2_O_3_ > uncatalysed. The results obtained were in agreement with the H_2_ yield and total gas yield analyses (Fig. 5a), implying that higher reducibility gave better H_2_ and other gaseous productions.

**Figure 6:**
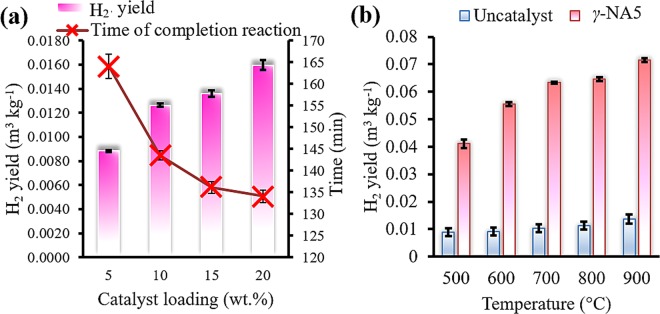
Effect of catalyst loading against H_2_ yield and time of completion reaction (**a**). [Reaction conditions: reaction temperature 800 °C, and flow rate 10 sccm], and Influence of temperature on hydrogen yield (**b**) [Reaction conditions: catalyst to biomass ratio 20 wt. % and flow rate 10 sccm].

**Table 2:** Effect of catalyst on product yields and gas composition.

Catalyst	Uncatalyst	Macroscopic *γ*-NA5
Gasifier temperature (°C)	900	900
Catalytic temperature (°C)	900	900
Gas yield (m^3^ kg^−1^)	0.0764	0.2601
**Gas composition**		
H_2_ content (Vol.%)	17.90	20.90
CO content (Vol.%)	43.66	47.59
CH_4_ content (Vol.%)	5.80	5.43
CO_2_ content (Vol.%)	32.64	26.08

Various effects Vs. Gas yield (m^3^ kg^-1^); and gas composition (vol% *i.e*. H_2_, CH_4_, CO_2_ and CO).

**Figure 7:**
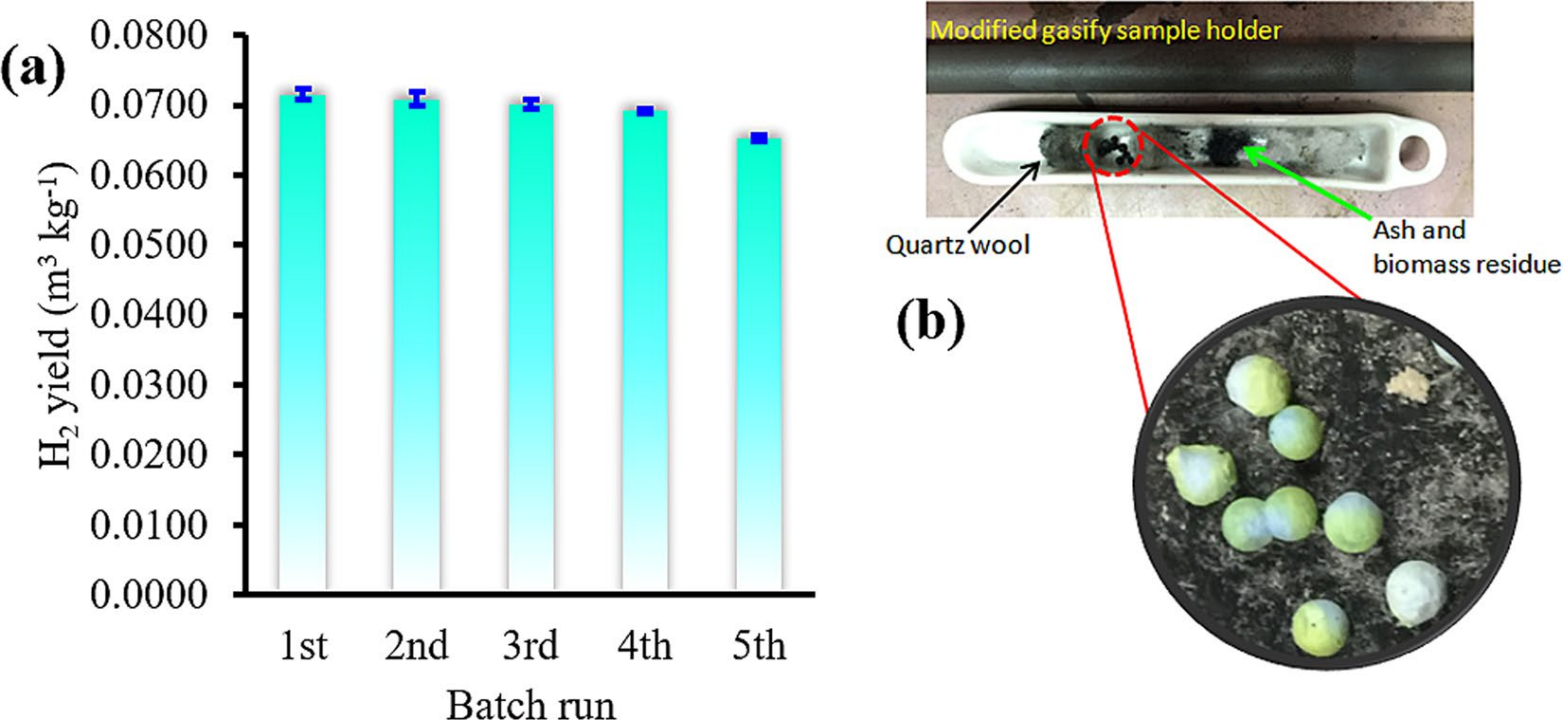
Reusability tests (**a**), and recyclable macroscopic *γ*-NA5 catalyst after every batch reaction (**b**).

Figure 6a demonstrates the effects of catalysts concentration over H_2_ yield and gasification completion time. It showed that a higher concentration of *γ*-NA5 catalyst to biomass ratio (up to 20 wt.%) did not merely improved its H_2_ yield (0.0160 m^3^ kg^−1^), but it also shortened the reaction completion duration (135 min). This is due to a higher concentration of catalyst that resulted in a thicker catalyst bed, which possesses ample accessible active sites for tar cracking of palm EFB biomass to take place. Comparatively, the H_2_ yields has been compromised when the catalyst loading was less than 20 wt.% (5, 10, and 15 wt.% produced H_2_ yields of 0.0088, 0.0126, and 0.0126 m^3^ kg^−1^, respectively).

Generally, the palm EFB biomass decomposes during partial oxidation process to gaseous products, i.e. H_2_, CO, CO_2_ CH_4_, and char, as the by-products. The equations below express the formation of gaseous and the consumption of carbon for methanation (1), water-gas shift reaction (2), dry reforming (3), methane reforming reaction (4), and Boudard reaction (5) in the biomass conversion^20,28,55^:

1 $${\rm{C}}+{{\rm{CO}}}_{2}\to 2\mathrm{CO}\Delta ^\circ {H}_{923}\,(\,-\,89.0\,{\rm{kJ}}\,{{\rm{mol}}}^{-1})$$

2 $${\rm{CO}}+{{\rm{H}}}_{2}{\rm{O}}\to {{\rm{CO}}}_{2}+{{\rm{H}}}_{2}\Delta ^\circ {H}_{923}\,(\,-\,35.6\,{\rm{kJ}}\,{{\rm{mol}}}^{-1})$$

3 $${{\rm{CH}}}_{4}+{{\rm{CO}}}_{2}\to 2{{\rm{H}}}_{2}+{\rm{CO}}\,\Delta ^\circ {H}_{923}\,(247.3\,{\rm{kJ}}\,{{\rm{mol}}}^{-1})$$

4 $${{\rm{CH}}}_{4}+{{\rm{H}}}_{2}{\rm{O}}\to {\rm{CO}}+3{{\rm{H}}}_{2}\,\Delta ^\circ {H}_{923}\,(225\,{\rm{kJ}}\,{{\rm{mol}}}^{-1})$$

5 $${\rm{C}}+{{\rm{H}}}_{2}{\rm{O}}\to 2{\rm{CO}}\,\Delta ^\circ {H}_{923}\,(171\,{\rm{kJ}}\,{{\rm{mol}}}^{-1})$$

The effectiveness of *γ*-NA5 catalyst in enhancing the H_2_ yield produced from palm EFB gasification reaction is significantly revealed in Fig. 6b. The composition of the produced gases is demonstrated in Fig. S4 and the data is summarised in Table 2. As illustrated in Fig. 6b, the uncatalysed gasification process (in the absence of *γ*-NA5 catalyst) renders poor gasification reaction, with a significantly low amount of hydrogen production than the catalytic reaction. In contrast, about 7-fold increment in H_2_ generation, especially at 900 °C was observed when 20 wt% catalyst was used. Theoretically, high syngas (H_2_ and CO) production is achievable in the initial devolatilisation step within the temperature range of 500 °C–750 °C, which is during dry reforming (4), methane reforming (5) reactions, and char and tar cracking. Clearly, according to the Le Chatelier’s principle, high temperature favours productions in endothermic reactions, which are during the methane reforming and tar cracking process^56^. To support the aforementioned statement, we confirmed that at a high temperature of 900 °C, in which the gas compositions of CH_4_ and CO_2_ were reduced, the production of syngas (H_2_ and CO) were enhanced, as observed in Fig. S4 and Table 2. This observation was in great agreement with the other experimental observations in literature^57^. The improved physico-chemical properties (stable Ni active phase and surface area) of macroscopic spherical *γ*-NA5 catalyst has significantly enhanced the tar cracking process at a higher temperature.

The recoverability, reversibility, and reusability of a catalyst is an important factor to be considered for large-scale industrial usage. The reusability of *γ*-NA5 catalyst was investigated, as shown in Fig. 7a and tabulated in Table 3. The used *γ*-NA5 catalyst separated from the reactant medium such as quartz wool and ash residues with ease (Fig. 7b), and subsequently reused for further batch reaction without requiring any pre-treatment. The following reaction batches were carried out under the same reaction conditions: reaction temperature of 900 °C, catalyst to biomass ratio of 20 wt.%, and flow rate of 10 sccm. The loading of 5% Ni on the alumina support has successfully suppressed the coke formation, thereby significantly enhanced the H_2_ production. Worth to be mentioned, the catalyst can be reused five times successively without compromising the H_2_ yield.

**Table 3:** Repeated gasification reaction of *γ*-NA5 catalyst.

Use batch	Yield of H_2_ (m^3^ kg^−1^)
First	0.0716
Second	0.0708
Third	0.0701
Forth	0.0692
Fifth	0.0654

Upon the fifth reaction batches, slight deactivation of the *γ*-NA5 catalyst was observed due to coke formation and also oxidation of active species on the catalyst surface. However, the active phase of macroscopic catalyst can be easily regenerated by a simple calcination process, which is highly suitable for industrial application. In contrast, the bulk NiO was regarded as a non-user friendly catalyst as it was not able to be recovered and reused after every batch reaction, due to the bulk NiO powder mixing with the biomass ash at the end of the gasification reaction.

**Figure 8:**
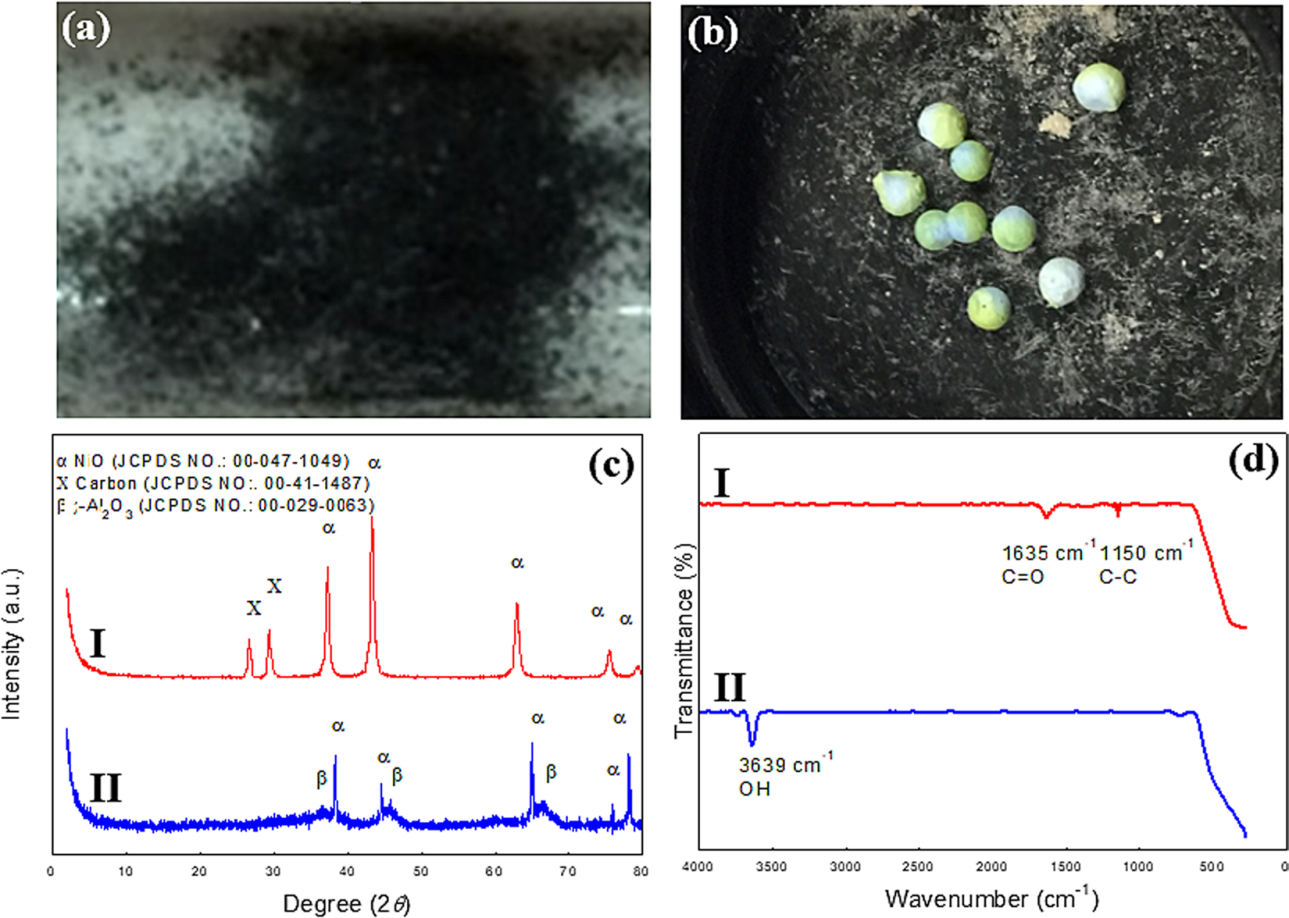
Post-reaction characterization of the used NiO (**a**) and macroscopic *γ*-NA5 catalyst (**b**) catalysts, XRD profile of spent NiO (**c-I**) and *γ*-NA5 (**c-II**) catalysts, and FTIR spectrum of used NiO (**d-I**) and *γ*-NA5 (**d-II**) catalysts.

Although NiO is cheap and easily available, the powdered NiO cannot be recovered and reused after batch reaction, due to the bulk NiO powder mixing with the biomass ash at the end of the gasification reaction^54^ (Fig. 8a). In addition, the high tendency towards carbon deposition is another obstacle to reuse the powdered NiO catalyst. Hence, no recovering performance of the powdered NiO catalysts was conducted in this study. We have also attempted to gain further the crystalline structure, surface functional property of the spent catalysts. The crystal structure and surface functional group was remained unchanged verifying the structural stability of used *γ*-NA5 catalyst (Figure c-II). The XRD showed peaks at 2*θ* values of 26.7° and 29.5°, both could be associated with the carbons (JCPDS File No. 00-41-1487)^58^ deposited on surface of used NiO catalyst. The FTIR peaks at 1635 cm^−1^ and 1150 cm^−1^ were assigned to the C-C and C=O vibrations, respectively which further confirming the coke deposition occurred in the used NiO catalyst^54^ (Fig. 8d-I). Thus, this macroscopic catalyst presented here opens good strategies for the production of hydrogen from biomass with excellent activity and durability catalyst toward clean H_2_ energy production.

## Conclusions

In conclusion, the loading of 20 wt% *γ*-NA5 catalyst accelerated the catalytic process and exhibited a high H_2_ production yield of 0.0716 m^3^ kg^−1^, which was about 5.6-fold and 1.3-fold higher than the singly *γ*-Al_2_O_3_ and NiO powder catalysts, respectively. The synergistic effects between *γ*-Al_2_O_3_ and NiO enhanced the reusability and reversibility of the *γ*-NA5 catalyst whereby 91% H_2_ production efficiency was still retained after five batches of gasification process. Interestingly, the distinctive features of *γ*-NA5 pellet from the conventional powder-like catalyst enhanced the separation of used catalyst and biomass ash, subsequently encouraging the regeneration process. It is worth mentioning that the utilisation of *γ*-NA5 pellet was first to be applied for syngas production and is envisioned to be the next promising catalyst for industrial use. In brief, the pellet formed *γ*-NA5 catalyst will be the next rising star in the gasification industrial process due to its cost effectiveness, environmental friendliness, ease of regeneration, and high catalytic, reusability, and reversibility properties.

## Experimental Section

### Materials

The feedstock material used in current study was palm EFB which obtained from a palm oil biorefinery factory in Selangor, Malaysia. The EFB feedstock was prepared and treated before being used in the gasification process. Initially, the raw feed containing high amount of moisture was air dried for 7 days until the moisture content reached to below 20%. The dried feed was then crashed and milled to become smaller size fibers with mean length of 2–5 mm, subsequently sieved to ≤250 μm of particle size distribution in fine powder. Ultimate analysis was carried out on a treated EFB sample to evaluate the elemental composition of the biomass. The obtained results and data analysis are presented in Table S1. Paraffin oil (density at 20 °C, *ρ* = 0.87 g ml^−1^) was obtained from Sigma-Aldrich, USA. Aluminium oxide hydroxide (AlOOH) powder was purchased from BASF chemical company (G-250), USA.

### Synthesis of macroscopic spherical particles

Macroscopic gamma-alumina (*γ*-Al_2_O_3_) support was synthesised by means of oil drop granulation approach^41^. Initially, 30 g of aluminium oxide hydroxide (AlOOH) powder were mixed with 100 mL of deionised water under stirring (500 rpm). The mixture was then homogenised with ultrasonic wave for 5 minutes at an amplitude of 70%. The mixture was added with concentrated hydrochloric acid until it formed a white gel-paste at pH ranged from 1.0–7.6. The ideal pH was determined from the homogeneity of suspended solution via gelling process and used to prepare the AlOOH suspension solution. All experiments were performed at room temperature of 25 °C. Subsequently, a peristaltic pump is used to transferred the gel into a liquid column. The experimental set-up was constructed to synthesise the particles as shown in Fig. S3. The gel droplets were formed and aged in the ammonia solution for 1 hour. The particles were separated, washed and then dried in air at room temperature (25 °C) for 12 h. Consequently, the dry and rigid particles were calcined at 800 °C for 5 h with the ramp at 5 °C min^−1^. The synthesised macroscopic *γ*-Al_2_O_3_ particles were keep nicely in a firmly sealed vacuum a desiccator to avoid contamination of moisture and impurities from atmosphere.

### Synthesis of macroscopic nickel supported catalyst

To synthesise macroscopic nickel supported catalyst, the calcined beads were then impregnated with an aqueous solution (each separated in 10 ml deionized water) of NiCl_2_•6H_2_O (nickel (II) chloride hexahydrate) under a pH control condition. For a typical method, 5 wt.% of Ni was doped onto the prepared macroscopic spherical support (5 g) via impregnation process by weighing appropriate amount of NiCl_2_ and dissolving it in 5 ml of deionised water. Ammonia solution was used to maintained the pH of solution at 8. The doped catalyst supports were dehydrated in an oven at 100 °C for 12 h to remove the water content. Finally, the bead-supported catalysts were calcined at 500 °C for 5 h to make the catalyst active for flash gasification reaction. The macroscopic nickel supported catalyst was donated as *γ*-NA5.

### Catalysts characterization

X-ray diffraction (XRD) patterns were carried out at ambient temperature with a Shimadzu diffractometer model XRD-6000 employing CuK*α* radiation at 40 kV and 30 mA made by Philips glass diffraction X-ray tube broad focus 2.7 kW. It performed over 2*θ* range between 10° and 80° at a scanning speed of 2° min^−1^.

H_2_–temperature programmed reduction measurement (TPR-H_2_) was achieved using Thermo-Finnigan TPD/R/O 1100 series equipped with thermal conductivity detector (TCD). Each of the samples (~0.1 g) was first pretreated under an N_2_ environment and analysis continued in a flow of 5% H_2_ in argon (25 mL min^−1^) in the temperature range 50–900 °C.

Brunauer-Emmett-Teller (BET) surface area was analysed by N_2_ adsorption/desorption at −196 °C using Thermo-Finnigan Sorpmatic 1990 series.

The shape and size measurement of the support beads were conducted by analysing the digital images apprehended by a digital camera (Moticam-350, version 2.0 ML, China) installed on a stereozoom microscope (Stemi DV4, Carl Zeiss, Germany) using image analyser (SigmaScan Pro 5.0, SPSS Inc). Sphericity factor (SF) of granule particle was computed and calculated from the following equation: SF = (D_MAX_ − D_VER_)/(D_MAX_ + D_VER_), where D_MAX_ is refer to maximum diameter passing via a granules centroid (mm) and D_VER_ is the diameter vertical to DMAX passing via the granule centroid in unit of mm. The overall diameter of granule particle, d_*p*_ (mm), is derived as following equation: d_*p*_ = *kk*_LF_(6*d*_T_γ/*ρg*)^1/3^ = K(6*d*_T_γ/*ρg*)^1/3^, where *g* is gravitational force (m s^−2^). In addition, four equations, i.e., *k*_g_ = *d*_g_/*d*_f_, *k*_a_ = *d*_a_/*d*_f_, *k*_c_ = *d*_c_/*d*f and *k*_LC_ = 0.98–0.04*d*_T_, where *k*_g_, *k*_a_ and *k*_c_ are referred to the shrinkage factor of granules after gelling, drying and calcinating, respectively, and *k*_LF_ is liquid lost factor. In contrast, *d*_f_, *d*_g_, *d*_a_ and *d*_c_ are attributed to the diameter of granules when dripping, after gelling, drying and calcinating, respectively, and *d*_T_ is the dimensionless tip diameter employed in equation of *d*_p_.

For the morphology analysis, scanning electron microscopy (SEM) was taken with a JOEL JSM6700F Field Scanning Electron Microscope (FESEM). To protect the induction of electric current, the catalyst was coated with Au (gold) by a sputter coater with maximum operating voltage used was 25 kV.

For the elementally mapping of microstructures investigation, energy dispersive X-ray (EDX) analysis was carried out by a SEM with Energy Dispersive X-ray Spectrometry (EDS). The main unit (FESEM) was coupled with the EDS, Rayny EDX-720 spectrometer in order to analyst the metal ion composition i.e., Ni, Al and O.

For particle size analysis, the distribution of particle was plotted from the SEM measurements by using ImageJ 1.46r, USA Image Processing and Analysis in Java Software. From the plotted histogram, value of mean diameter and standard deviation of particle NiO can be calculated.

FTIR (Fourier transform-infrared) spectroscopy by PerkinElmer (PC) spectroscopy spectrum 100 FTIR spectrometer, wavenumber ranged from 400–4000 cm^−1^ was performed to verifying the surface moiety attaching on the catalyst,

### Hydrogen production via fast gasification reaction

**Scheme 4:**
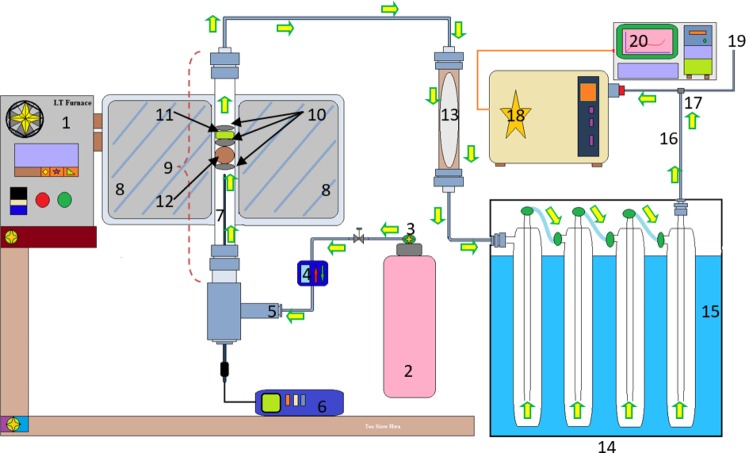
Steel piping reactor setup. Furnace temperature controller (1); 5% oxygen: 95% helium (2); pressure gas controller (3); flow controller (10 sccm) (4); gas inlet (5); thermocouple reader (6); K-type thermocouple (7); heating zone of furnace (8); steel piping of reactor (9); quartz wool (10); catalyst (11); biomass (12); cotton wool filter (13); cold trap (14); ice bath (15); outlet leading towards quadrupole mass spectrometer (QMS) (16); T-gas splitter (17); standalone quadrupole mass spectrometer (18); outlet gases to atmosphere (19) and data analysis (20).

0.3 g of oil palm empty fruit bunches powder together with 0.06 g of catalyst sample was loaded into the stainless steel piping setup as shown in Scheme 4. Quartz wool was used to separate the catalyst from the EFB and also to act as a support for the EFB in the stainless steel piping. The glass wool also prevents the catalyst from being pushed char that are produced during the reaction.

A cold trap immersed in an ice bath was used to condense any tar or volatile compound formation from the product gas before analysis. 10 standard cubic centimeters per minute (sccm) of 5% O_2_ diluted in He was flowed through the stainless steel piping setup in an updraft orientation. The 5% O_2_ gas diluted in He was allowed to flow through the stainless steel piping for 10 minutes prior to starting the reaction to ensure that the atmosphere inside the piping only contains the desired reaction gas. The outlet of the stainless steel piping setup was then connected to an online quadrupole mass spectrometer. The furnace was preheated to 900 °C and the stainless steel piping setup was then swiftly placed into the furnace for isothermal heating. The reaction carried out for an hour and the gas produced was analysed in real time using the online quadrupole mass spectrometer (QMS). The catalytic performance test was conducted with the synthesised catalyst including commercial bulk NiO using the following parameters *i.e*. gasification temperature of 900 °C, flow rate of 10 sccm, and biomass-catalyst ratio of 20% wt. The uncatalysed reaction was also conducted for comparison purpose. The catalyst that demonstrated high H_2_ yield was then selected for further analysis in order to optimise the influence of catalyst loading for H_2_ production. Finally, the reusability tests were performed with optimised conditions.

## Supplementary information

Supplementary information 1–3
