# Supplementary material for: Facile Recoverable and Reusable Macroscopic Alumina Supported Ni-based Catalyst for Efficient Hydrogen Production
Source: Sci Rep. 2019 Nov 8;9:16358. doi: 10.1038/s41598-019-52857-4 (PMC6841685; doi:10.1038/s41598-019-52857-4)
Supplement: Supplementary file 1 — Supplementary information 1–3 [file 41598_2019_52857_MOESM1_ESM.pdf]

Supplementary Information

Facile Recoverable and Reusable Macroscopic Alumina Supported Ni-based Catalyst for Efficient Hydrogen Production

Siow Hwa Teo[1,2,*], Davin Kin Yew Yap[1,2], Nasar Mansir[1,2,], Aminul Islam[1,4] & Yun Hin Taufiq-Yap[1,2,3*]

[1]Catalysis Science and Technology Research Centre, Faculty of Science, Universiti Putra Malaysia, 43400, UPM Serdang, Selangor, Malaysia

[2]Department of Chemistry, Faculty of Science, Universiti Putra Malaysia, 43400 UPM, Serdang, Selangor, Malaysia

[3]Chancellery Office, Universiti Malaysia Sabah, 88400 Kota Kinabalu, Sabah, Malaysia

[4]Department of Petroleum and Mining Engineering, Jashore University of Science and Technology, Jashore, 7408, Bangladesh

*corresponding and requests for materials should be addressed to S.H.T. (email: siowhwa_teo@hotmail.com) or Y.H.T.Y. (email: taufiq@upm.edu.my)

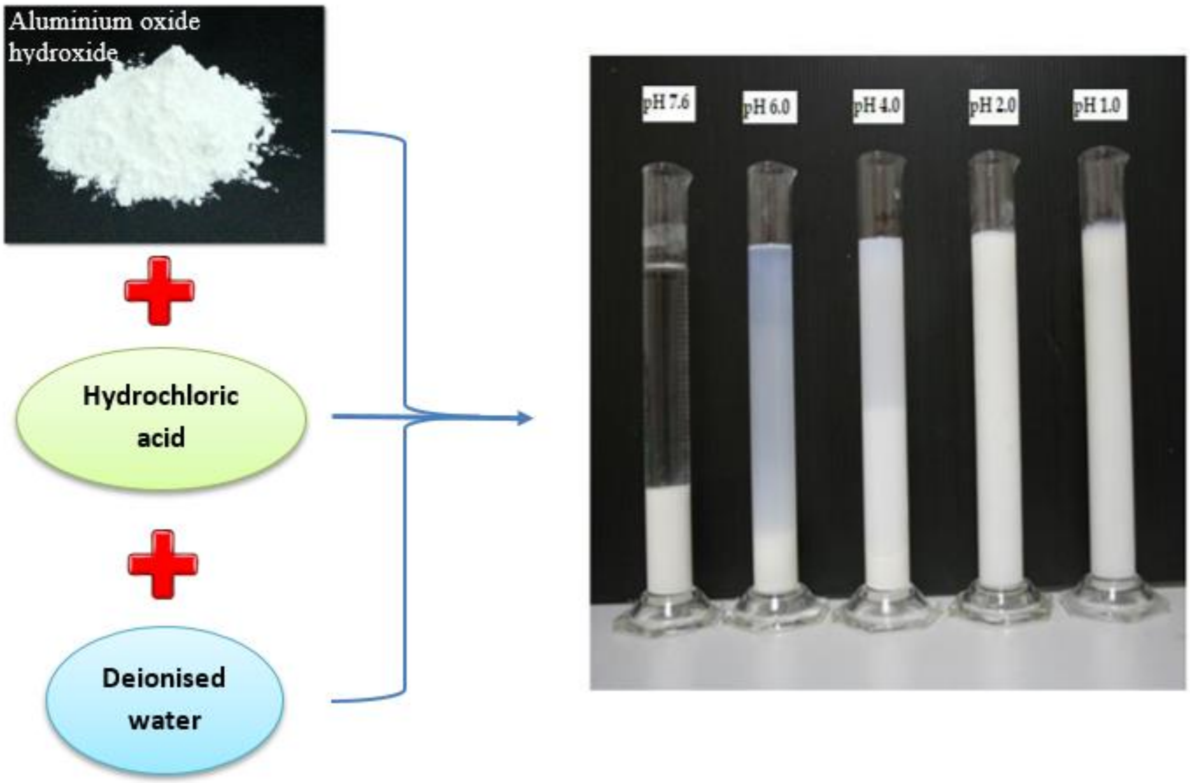

Figure S1. The gelling property of aluminium oxide hydroxide in various pH values ranged 1.0 – 7.6.

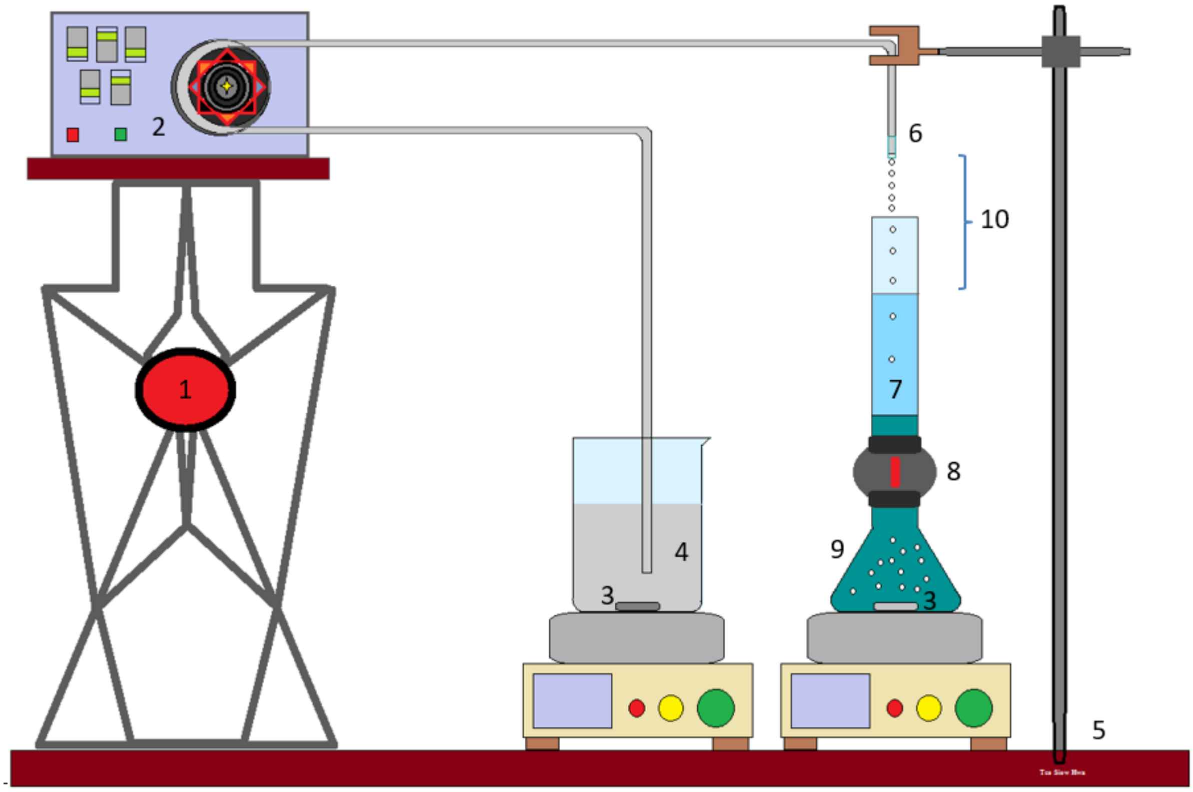

Figure S2. Experimental set-up for production of macroscopic particles *i.e.* rinder (1); peristaltic pump with pressure of 0.4 mL min^{-1} (2); magnetic stirrer (3); boehmite gel (4); retort stand (5); needle (6); 10 mL oil layer in a liquid column (7); stopcock (8); 15 mL ammonia solution (9); 5 cm collecting distance (10).

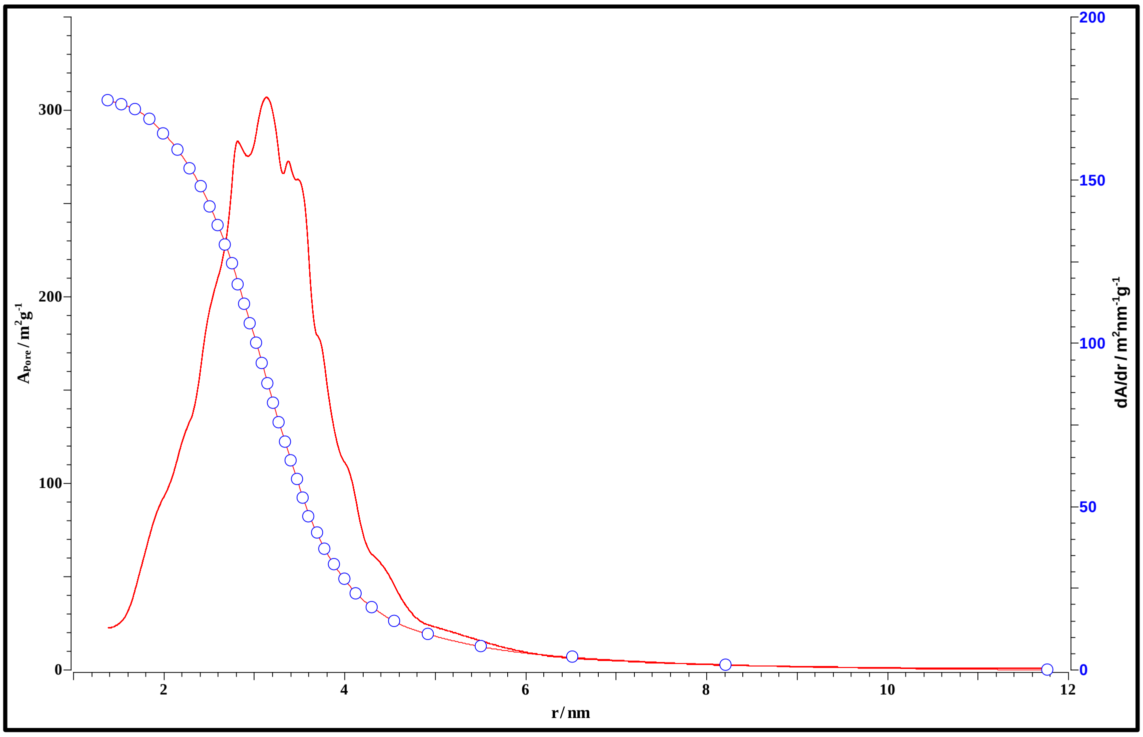

Figure S3. The BJH pore size distribution of the macroscopic γ-NA5 catalyst

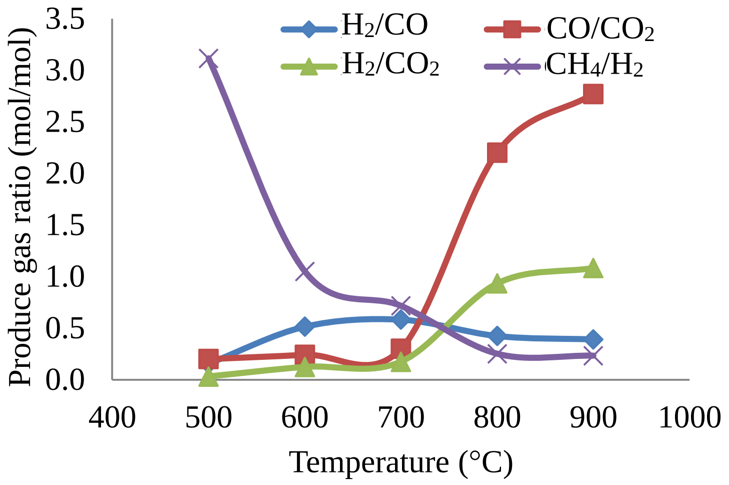

Figure S4. Composition of the produced gases [Reaction conditions: reaction temperature 900 °C, catalyst to biomass ratio 20 wt. % and flow rate 10 sccm]

Table S1. Elemental composition of the biomass

Material	Elemental composition				
	C (wt.%)	H (wt.%)	N (wt.%)	O* (wt.%)	Ash (wt.%)
	39.44	6.02	1.39	50.02	3.17

* By difference
